# The effects of probiotic and selenium co-supplementation on parameters of mental health, hormonal profiles, and biomarkers of inflammation and oxidative stress in women with polycystic ovary syndrome

**DOI:** 10.1186/s13048-018-0457-1

**Published:** 2018-09-14

**Authors:** Mehri Jamilian, Shirin Mansury, Fereshteh Bahmani, Zahra Heidar, Elaheh Amirani, Zatollah Asemi

**Affiliations:** 1 Department of Gynecology and Obstetrics, Endocrinology and Metabolism Research Center, School of Medicine, Arak University of Medical Sciences, Arak, Iran; 2 Research Center for Biochemistry and Nutrition in Metabolic Diseases, Kashan University of Medical Sciences, Kashan, I.R. Iran; 3 Infertility and Reproductive Health Research Center, Shahid Beheshti University of Medical Sciences, Tehran, Iran

**Keywords:** Probiotic, Selenium, Mental health, Hormonal profiles, Inflammatory markers, Polycystic ovary syndrome

## Abstract

**Background:**

The aim of this study was to evaluate the effect of the co-administration of probiotic and selenium on parameters of mental health, hormonal profiles, and biomarkers of inflammation and oxidative stress in women with PCOS. Data on the effects of selenium and probiotic co-supplementation on mental health, hormonal and inflammatory parameters of patients with polycystic ovary syndrome (PCOS) are scarce. This investigation was carried out to evaluate the effects of selenium and probiotic co-supplementation on mental health, hormonal and inflammatory parameters in women with PCOS.

**Methods:**

This randomized, double-blinded, placebo-controlled clinical trial was conducted on 60 subjects, aged 18–40 years old. Participants were randomly allocated into two groups to intake 8 × 10^9^ CFU/day probiotic plus 200 μg/day selenium supplements (*n* = 30) or placebo (*n* = 30) for 12 weeks. Hormonal and inflammatory parameters were measured at baseline and after the 12-week intervention.

**Results:**

Probiotic and selenium co-supplementation resulted in a significant improvement in beck depression inventory (β − 0.76; 95% CI, − 1.26, − 0.26; *P* = 0.003), general health questionnaire scores (β − 1.15; 95% CI, − 1.97, − 0.32; *P* = 0.007) and depression anxiety and stress scale scores (β − 1.49; 95% CI, − 2.59, − 0.39; *P* = 0.009) compared with the placebo. Furthermore, probiotic and selenium co-supplementation significantly reduced total testosterone (β − 0.26 ng/mL; 95% CI, − 0.51, − 0.02; *P* = 0.03), hirsutism (β − 0.43; 95% CI, − 0.74, − 0.11; *P* = 0.008), high-sensitivity C-reactive protein (hs-CRP) (β − 0.58 mg/L; 95% CI, − 0.97, − 0.19; *P* = 0.004) and *malondialdehyde (MDA)* levels (β − 0.29 μmol/L; 95% CI, − 0.56, − 0.02; P = 0.03), and significantly increased total antioxidant capacity (TAC) (β + 84.76 mmol/L; 95% CI, + 48.08, + 121.44; *P* < 0.001) and total glutathione (GSH) levels (β + 26.78 μmol/L; 95% CI, + 4.33, + 49.23; *P* = 0.02) compared with the placebo.

**Conclusions:**

Overall, the co-administration of probiotic and selenium for 12 weeks to women with PCOS had beneficial effects on mental health parameters, serum total testosterone, hirsutism, hs-CRP, TAC, GSH and MDA levels.

This study was prospectively registered in the Iranian website (www.irct.ir) for registration of clinical trials (http://www.irct.ir: IRCT20170513033941N22).

**Trial registration:**

IRCT20170513033941N22.

## Background

Polycystic ovary syndrome (PCOS) is an endocrine disturbances accompanied by a lot of conditions as well as economic burden [1]. Excess of androgens and related consequences including hirsutism and acne are notable clinical features of this disorder [2]. Prior evidence indicated that defected antioxidant defense and elevated inflammatory status contribute to the progression of PCOS [3, 4]. In addition, it has been stated that the gut microbiota dysbiosis is involved in the pathogenesis of PCOS [5, 6]. A lower concentration of selenium is also reported in women with PCOS and a negative correlation is illustrated between selenium and testosterone levels [7]. In these patients, life style modification, as the first line of treatment, is associated with improvement in body composition, insulin resistance, hyperandrogenism and clinical manifestations [8].

Previous studies have shown several beneficial effects of probiotic supplementation on glycemic control in PCOS patients [9, 10]. Moreover, some researchers have demonstrated that probiotic consumption in patients with osteoarthritis [11], type 2 diabetes mellitus (T2DM) [12], and gestational diabetes mellitus (GDM) [13] was associated with decreased inflammatory markers. On the other hand, selenium supplementation has been reported to improve antioxidant parameters in patients with metabolic syndrome [14]. In addition, in a study by Abedelahi et al. [15], it was seen that sodium selenite improved the in vitro follicular development by increasing total antioxidant capacity (TAC) levels. Selenium supplementation is thought to confer protective effects against oxidative stress and inflammation through reducing the formation of reactive oxygen species (ROS) and modulating of cellular signaling pathways [16]. Probiotic may affect antioxidant status and hormonal profiles by alleviating insulin resistance and anti-inflammatory properties [17, 18]. Recently, it is stated that probiotic plus selenium co-administration in animal models give better effects on metabolic responses and reproductive performance thorough additive actions [19, 20]. Combined probiotic and selenium supplementation may ameliorate clinical symptoms of patients with PCOS by improving their metabolic profiles and attenuating oxidative stress and inflammation. Therefore, we conducted this study to determine the effects of probiotic and selenium co-supplementation on parameters of mental health, hormonal profiles, and biomarkers of inflammation and oxidative stress in women with PCOS.

## Methods

This randomized double-blinded, placebo-controlled trial registered in the Iranian website for registration of clinical trials (http://www.irct.ir: IRCT20170513033941N22) and followed the Declaration of Helsinki and Good Clinical Practice guidelines. This trial was conducted among 60 women with PCOS, diagnosed based on the Rotterdam criteria [21], aged 18–40 years old whom referred to the Kosar Clinic in Arak, Iran, between December and March 2018. The study protocol was approved by the Ethics Committee of Arak University of Medical Sciences (AUMS). Written informed consent was obtained from all participants prior to the intervention. Exclusion criteria were as follows: pregnancy, adrenal hyperplasia, androgen-secreting tumors, hyperprolactinemia, thyroid dysfunction, diabetes at enrollment.

### Supplementation

Sixty PCOS women were randomized into two groups to intake either 8 × 10^9^ CFU/day probiotic containing *Lactobacillus acidophilus*, *Lactobacillus reuteri*, *Lactobacillus fermentum* and *Bifidobacterium bifidum* (2 × 10^9^ CFU/g each) plus 200 μg/day selenium (*n* = 30) or placebo (*n* = 30) for 12 weeks. Shape and size of supplements and placebos capsules were similar and manufactured by Familact (Tehran, Iran) and Barij Essence Pharmaceuticals (Kashan, Iran), respectively. Compliance with probiotic and selenium supplements and the placebos was examined by asking subjects to return the medication containers and through brief daily cell phone reminders to take the supplements. All subjects completed 3-day diet recall at weeks 0, 3, 6, 9 and 12 of the intervention. Daily macro- and micro-nutrient intakes were calculated by nutritionist IV software (First Databank, San Bruno, CA).

### Assessment of outcomes

Hormonal profiles were considered as the primary outcomes. Mental health parameters, biomarkers of inflammation and oxidative stress were recognized as the secondary outcomes.

### Clinical measures

Mental health was judged with beck depression inventory (BDI), general health questionnaire-28 (GHQ-28) and depression anxiety and stress scale (DASS) at baseline and week 12 of the intervention. BDI is a self-compiled questionnaire of 21 items in multiple choice format [22]. The GHQ-28 comprises 28-item consisting of 4 subscales [23]. DASS questionnaire consists of three 14-item self-report scales that measure depression, anxiety and stress [24].

### Biochemical assessment

Fasting blood samples (15 ml) were collected at baseline and the end of the intervention at Arak reference laboratory. Serum total testosterone and sex hormone-binding globulin (SHBG) with inter- and intra-assay with inter- and intra-assay CVs of lower than 7%% were measured using commercial validated kits (DiaMetra, Milano, Italy). Free androgen index (FAI) was calculated as the percentage of total testosterone/SHBG. Serum high sensitivity C-reactive protein (hs-CRP) concentrations were quantified using commercial ELISA kit (LDN, Nordhorn, Germany) with inter- and intra-assay CVs of lower than 7%. The plasma NO using Griess method [25], TAC concentrations using Benzie and Strain method [26], total glutathione (GSH) using Beutler method [27] and malondialdehyde (MDA) concentrations thiobarbituric acid reactive substances spectrophotometric test [28] were quantified with CVs less than 5%.

### Statistical analyses

The Kolmogorov-Smirnov test was done to determine the normality of data. Differences in dietary intakes between treatment groups were detected with independent-sample *t*-tests. Multiple linear regression models were used to assess the treatment effects on study outcomes after adjusting for confounding parameters including; age, and BMI. Significance of the treatment effects was presented as the mean differences with 95% confidence interval. Bootstrapping was also used as a sensitivity analysis of confidence interval. *P*-values < 0.05 were considered statistically significant. All statistical analyses were done using the Statistical Package for Social Science version 18 (SPSS Inc., Chicago, Illinois, USA).

## Results

**Fig. 1:**
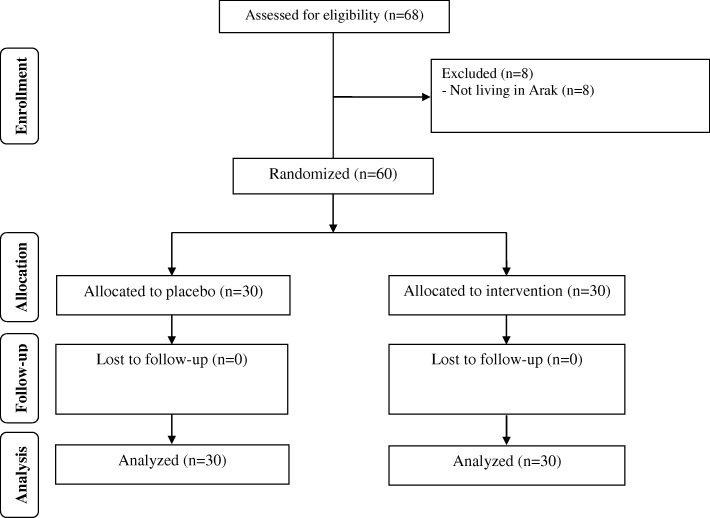
Summary of patient flow diagram

As demonstrated in the study flow diagram (Fig. 1)**,** during the enrollment phase of the study, there were 68 women with PCOS; however, 8 participants did not meet the inclusion criteria and thus were excluded. During the follow-up, 60 participants [placebo (*n* = 30) and probiotic and selenium co-supplementation (*n* = 30)] completed the trial.

Mean age, height, weight and BMI at baseline and after the 12-week intervention were not statistically different between the two groups (Table 1)**.**

**Table 1:** General characteristics of study participants

	Placebo group (*n* = 30)	Probiotic plus selenium group (*n* = 30)	P^a^
Age (y)	25.6 ± 3.8	26.0 ± 5.3	0.71
Height (cm)	161.6 ± 4.7	161.0 ± 4.4	0.63
Weight at study baseline (kg)	63.4 ± 7.7	63.9 ± 9.3	0.79
Weight at end-of-trial (kg)	63.2 ± 7.6	63.5 ± 9.2	0.86
Weight change (kg)	−0.2 ± 0.7	−0.4 ± 0.5	0.23
BMI at study baseline (kg/m^2^)	24.0 ± 3.0	24.6 ± 3.3	0.67
BMI at end-of-trial (kg/m^2^)	24.2 ± 3.0	24.5 ± 3.3	0.73
BMI change (kg/m^2^)	−0.1 ± 0.3	−0.2 ± 0.2	0.23

Data are means± SDs

^a^Obtained from independent-samples *t*-test

There was no statistically significant difference in terms of dietary macro- and micro-nutrient intakes between probiotic plus selenium, and placebo groups (Data not shown).

Probiotic and selenium co-supplementation resulted in a significant improvement in BDI (β − 0.76; 95% CI, − 1.26, − 0.26; *P* = 0.003), GHQ (β − 1.15; 95% CI, − 1.97, − 0.32; *P* = 0.007) and DASS (β − 1.49; 95% CI, − 2.59, − 0.39; *P* = 0.009) compared with the placebo (Table 2). Furthermore, probiotic and selenium co-supplementation significantly reduced total testosterone (β − 0.26 ng/mL; 95% CI, − 0.51, − 0.02; *P* = 0.03), hirsutism (β − 0.43; 95% CI, − 0.74, − 0.11; *P* = 0.008), hs-CRP (β − 0.58 mg/L; 95% CI, − 0.97, − 0.19; *P* = 0.004) and *MDA* levels (β − 0.29 μmol/L; 95% CI, − 0.56, − 0.02; P = 0.03), and significantly increased TAC (β + 84.76 mmol/L; 95% CI, + 48.08, + 121.44; *P* < 0.001) and GSH levels (β + 26.78 μmol/L; 95% CI, + 4.33, + 49.23; *P* = 0.02) compared with the placebo.

**Table 2:** Mental health and metabolic profiles at baseline and after the 12-week intervention in women with polycystic ovary syndrome that received either probiotic plus selenium supplements or placebo^a^

Variables	Placebo group (*n* = 30)		Probiotic plus selenium group (*n* = 30)		Difference in outcome measures between probiotic plus selenium and placebo groups^a^	
	Baseline	Week 12	Baseline	Week 12	β (95% CI)	P^b^
BDI total scores	15.6 ± 4.4	15.3 ± 4.7	15.5 ± 4.0	14.4 ± 3.7	−0.76 (−1.26, − 0.26)	0.003
GHQ scores	45.4 ± 8.1	44.6 ± 8.2	44.2 ± 7.3	42.4 ± 6.7	−1.15 (− 1.97, −0.32)	0.007
DASS scores	86.3 ± 13.1	85.1 ± 12.9	85.1 ± 11.2	82.5 ± 10.9	−1.49 (−2.59, −0.39)	0.009
Total testosterone (ng/mL)	1.3 ± 0.5	1.3 ± 0.4	1.4 ± 0.7	1.1 ± 0.6	−0.26 (− 0.51, − 0.02)	0.03
SHBG (nmol/L)	40.3 ± 17.5	40.4 ± 18.3	47.1 ± 19.7	49.5 ± 22.1	1.82 (−1.77, 5.42)	0.31
mF-G scores	13.0 ± 3.7	13.0 ± 3.6	14.5 ± 3.2	14.0 ± 2.9	−0.43 (−0.74, − 0.11)	0.008
hs-CRP (mg/L)	2.5 ± 1.6	2.7 ± 1.5	2.4 ± 1.5	2.0 ± 1.5	−0.58 (− 0.97, − 0.19)	0.004
NO (μmol/L)	36.5 ± 3.8	36.8 ± 4.0	36.6 ± 2.3	37.8 ± 3.5	0.94 (−0.65, 2.54)	0.24
TAC (mmol/L)	909.9 ± 110.8	909.4 ± 122.7	933.3 ± 59.8	1012.9 ± 69.4	84.76 (48.08, 121.44)	< 0.001
GSH (μmol/L)	496.4 ± 88.1	497.8 ± 88.3	528.3 ± 88.3	552.9 ± 83.1	26.78 (4.33, 49.23)	0.02
MDA (μmol/L)	2.4 ± 0.5	2.6 ± 0.7	2.8 ± 0.2	2.5 ± 0.2	−0.29 (−0.56, −0.02)	0.03

Data are mean ± SDs

^a^“Outcome measures” refers to the change in values of measures of interest between baseline and week 12. β [difference in the mean outcomes measures between treatment groups (probiotic plus selenium group = 1 and placebo group = 0)]

^b^Obtained from multiple regression model (adjusted for baseline values of each biochemical variables, age and baseline BMI)

*BDI* beck depression inventory, *DASS* depression anxiety and stress scale, *GHQ* general health questionnaire, *GSH* total glutathione, *hs-CRP* high-sensitivity C-reactive protein, *mF-G* modified Ferriman Gallwey, *MDA* malondialdehyde, *NO* nitric oxide, *SHBG* sex hormone-binding globulin, *TAC* total antioxidant capacity

## Discussion

In the present study, for the first time, we evaluated the effects of combined probiotic and selenium supplementation on hormonal responses, biomarkers of inflammation and oxidative stress in women with PCOS. We found that the co-administration of probiotic and selenium for 12 weeks to women with PCOS had beneficial effects on mental health parameters, serum total testosterone, hirsutism, hs-CRP, TAC, GSH and MDA levels.

### Effects on hormonal profiles

Previous evidence showed that total- free testosterone, and other androgens are significantly increased in women with PCOS [29], which associate with further consequences including hirsutism, acne, and alopcia and predisposing to infertility in long term [30, 31]. Androgen excess also increases obesity, insulin resistance and blood pressure, which in turn contributes to developing cardiovascular disease [32]. In addition, it is stated that the reduction of testosterone levels improves endothelial dysfunction, body weight, and dyslipidemia and insulin sensitivity in these patients [33]. Our findings demonstrated that probiotic and selenium co-administration to women with PCOS decreased mF-G scores and total testosterone, but did not affect SHBG concentrations. In addition, co-supplementation significantly improved mental health parameters. There are few studies investigating the effects of only probiotic or selenium supplementation on hormonal features of women with PCOS. In line with the present study, our previous research indicated that 12-week synbiotic supplementation decreased mF-G scores and increased SHBG levels in women with PCOS [34]. Moreover, probiotic supplementation for 12 weeks to women with PCOS led to a significant improvement in hirsutism, total testosterone and SHBG values [35]. In addition, it is reported that an 8-week selenium supplementation in women with PCOS decreased mF-G scores, but did not affect free testosterone concentrations [36]. Although, selenium supplementation for 12 weeks did not affect total testosterone and SHBG levels in women with PCOS [37]. In contrast to our findings, probiotic supplementation to postmenopausal women did not enhance testosterone and SHBG levels [38]. The speculated mechanisms by which probiotic may improve hormonal profiles result from the balance of intestinal microbiota, enhancement of digestion and absorption of dietary nutrients [20], increasing insulin sensitivity [39], and interaction with the gut-brain axis [40]. In addition, induced oxidative stress involves in the development of hyperandrogenoism in PCOS [41]. The potential impacts of selenium supplementation on hormonal parameters may be explained by decreasing ROS production and elevating enzymatic anti oxidant activity [42].

### Effects on biomarkers of inflammation and oxidative stress

Previous studies indicated that PCOS subjects are at risk for elevated inflammatory markers and abnormal antioxidant defense [43, 44]. In addition, it is demonstrated that in women with PCOS, inflammation involves in pancreatic beta cell dysfunction, insulin resistance, atherogenesis and ovarian disturbance [45], which accelerated by anti-oxidant imbalance [46]. The correction of oxidative stress and inflammatory status lead to alleviated hyperandrogenemia and atherogenic profiles [47, 48]. Our study indicated that probiotic and selenium co-supplementation to patients with PCOS resulted in a significant reduction in hs-CRP and MDA levels, and a significant increase in TAC and NO, but unchanged GSH concentrations. Similarly, selenium enriched probiotic in few animal studies enhanced antioxidant status. For instance, it is observed that a 4-week probiotic and selenium co-administration to mice fed a high-fat diet led to a significant decrease in MDA levels [19]. Similar results were observed in piglets grown in high ambient temperature after 6 weeks taking probiotic and selenium combination [49]. The findings of the present study are in agreement with our previous study indicated that a 10-week selenium supplementation to pregnant women at risk for intrauterine growth restriction resulted in decreased hs-CRP and elevated TAC levels [2]. Furthermore, an 8-week probiotic intervention in women with GDM significantly improved inflammation and oxidative stress biomarkers [50]. Although, Shoaei et al. [10] did not find any significant change in hs-CRP levels among women with PCOS receiving probiotic supplements for 8 weeks. In contrast to our study, 3-month selenium supplementation in patients with T2DM did not affect TAC levels [51]. Probiotic may attenuate inflammation and oxidative stress through metal ion chelating ability, modification of inflammatory signaling pathways, producing antioxidant metabolites, upregulating the antioxidant activity of the host and downregulating ROS producing enzymes [52]. Moreover, selenium involves in antioxidant defense system and play important roles in the increasing of glutathione peroxidase (GPx) activity, decreasing nuclear factor-kappaB (NF-κB) activation, inhibiting MAP kinase pathways and altering the metabolism of arachidonic acid, which in turn result in anti-inflammatory effects [53].

The current study had few limitations. Due to limited funding, we were unable to determine the effects of probiotic and selenium co-supplementation on circulating selenium levels. In the current study, sample size was low and did not meet our expectation. Future studies with longer duration of intervention, and large sample size are required to confirm the validity of our findings. The other limitation of this study was that the group of women surveyed was highly heterogeneous (18–40 years). As we recruited patients with PCOS from a referral center, all patients were received similar treatments based on available guidelines. However, we believe that age range would not influence our findings because mean age was not significantly different between intervention and non-intervention groups. This should be taken into account in the interpretation of our findings.

## Conclusions

Overall, the co-administration of probiotic and selenium for 12 weeks to women with PCOS had beneficial effects on mental health parameters, serum total testosterone, hirsutism, hs-CRP, TAC, GSH and MDA levels.

## Abbreviations

BDI: Beck depression inventory
DASS: Depression anxiety and stress scale
GHQ: General health questionnaire
GSH: Total glutathione
hs-CRP: High-sensitivity C-reactive protein
MDA: Malondialdehyde
mF-G: modified Ferriman Gallwey
NO: nitric oxide
SHBG: sex hormone-binding globulin
TAC: total antioxidant capacity
